# Factors Influencing the Intracellular Concentrations of the Sofosbuvir Metabolite GS-331007 (in PBMCs) at 30 Days of Therapy

**DOI:** 10.3390/ph15030355

**Published:** 2022-03-15

**Authors:** Jessica Cusato, Lucio Boglione, Amedeo De Nicolò, Gian Paolo Caviglia, Simone Mornese Pinna, Alessia Ciancio, Giulia Troshina, Antonina Smedile, Miriam Antonucci, Valeria Avataneo, Alice Palermiti, Jacopo Mula, Alessandra Manca, Giuseppe Cariti, Marco Cantù, Giorgio Maria Saracco, Giovanni Di Perri, Antonio D’Avolio

**Affiliations:** 1 Department of Medical Sciences, Amedeo di Savoia Hospital, University of Turin, 10149 Turin, Italy; amedeo.denicolo@unito.it (A.D.N.); gianpaolo.caviglia@unito.it (G.P.C.); simone.mornesepinna@unito.it (S.M.P.); alessia.ciancio@unito.it (A.C.); giulia.troshina@gmail.com (G.T.); antonina.smedile@unito.it (A.S.); miriam.antonucci20@gmail.com (M.A.); valeria.avataneo@unito.it (V.A.); alice.palermiti@unito.it (A.P.); jacopo.mula@unito.it (J.M.); alessandra.manca@unito.it (A.M.); gcariti@hotmail.com (G.C.); giorgiomaria.saracco@unito.it (G.M.S.); giovanni.diperri@unito.it (G.D.P.); antonio.davolio@unito.it (A.D.); 2 Department of Traslational Medicine, University of Eastern Piedmont, 28100 Novara, Italy; lucio.boglione@med.uniupo.it; 3 Laboratory of Clinical Biochemistry and Pharmacology, Department of Laboratory Medicine EOLAB, Ente Ospedaliero Cantonale, CH-6500 Bellinzona, Switzerland; marco.cantu@eoc.ch; 4 Interdepartmental Center for Clinical and Experimental Pharmacology (CIFACS), University of Turin, 10149 Turin, Italy

**Keywords:** DAAs, single nucleotide polymorphism, pharmacokinetics, *ABCB1*, *ABCG2*, *HNF4*α

## Abstract

Sofosbuvir (SOF) is an HCV NS5B polymerase inhibitor, and GS-331007 is its major metabolite. The aim of this study was to investigate whether clinical and pharmacological factors could influence GS-331007 intracellular (IC) concentrations in peripheral blood mononuclear cells (PBMCs) associated with a sustained virological response in patients treated with SOF and ribavirin (RBV). Drug levels were analyzed using liquid chromatography at different days of therapy, whereas variants in genes encoding transporters and nuclear factors were investigated using real-time PCR. This study enrolled 245 patients treated with SOF; 245 samples were analyzed for pharmacogenetics and 50 were analyzed for IC pharmacokinetics. The GS-331007 IC concentration at 30 days was associated with its plasma concentration determinate at 30, 60 and 90 days of SOF-therapy and with daclatasvir concentrations at 7 days of therapy. No genetic polymorphism affected IC exposure. In linear multivariate analysis, ledipasvir treatment, baseline albumin and estimated glomerular filtration rate were significant predictors of IC exposure. This study presents data on an IC evaluation in a cohort of patients treated with SOF, also considering pharmacogenetics. These results could be useful for regions where SOF–RBV treatment is considered the standard of care; moreover, they could further deepen the knowledge of IC exposure for similar drugs in the future.

## 1. Introduction

For several years, the combination of pegylated-interferon (peg-IFN) and ribavirin (RBV) was the standard of care for chronic hepatitis C (CHC) therapy, but anemia was present as a side effect. Protease inhibitors were added to RBV and peg-IFN in 2011: boceprevir, telaprevir and simeprevir were developed. They had poor activity against HCV genotypes 2 and 3 and against the minor frequency genotypes 4, 5 and 6. Nevertheless, this triple therapy improved the response rate to 75%. In 2013, there was an important advance with the approval of sofosbuvir (SOF): a specific RNA polymerase inhibitor. It increased the response rate when combined with RBV and peg-IFN but led to an interferon-free treatment.

In the last few years, all-oral anti-HCV drugs reaching a response rate of 98% were approved. It is important to highlight that the therapy required only 8 to 12 weeks of treatment in most patients and was extremely well tolerated. These all-oral regimens revolutionized the treatment of hepatitis C: these therapies decreased the morbidity and mortality of this disease, leading to a reduction in cirrhosis and hepatocellular carcinoma worldwide [1,2,3].

Among the anti-HCV direct-acting antiviral drugs (DAAs), SOF is one of the most commonly used [4]. It is a nonstructural 5B polymerase inhibitor, used in combination with daclatasvir (DAC), ledipasvir (LDV), simeprevir (SIM) and RBV; however, RBV remains recommended for patients infected with genotype 2 HCV, as well as for older men and cirrhotic subjects [5,6,7,8]. SOF is a low-molecular-weight pro-drug. Food can increase the median exposure by >2-fold, and the oral assumption bioavailability in dogs is about 9% [9]. It consists of carboxyl esterase (CES) 1, histidine triad nucleotide-binding protein (HINT) 1, P-glycoprotein (P-gp, encoded by *ABCB1* gene) and breast cancer resistance protein (BCRP, encoded by the *ABCG2* gene) as a substrate. SOF is intracellularly metabolized into different metabolites: among these, GS-331007 is active, excreted in urine and retains nearly 90% of the total amount [10,11,12]. SOF undergoes several steps to produce pharmacologically active nucleotide metabolites: in the hepatocytes, CES1 cleaves SOF to produce metabolite X (high first-pass hepatic extract, about 70%). Then, phosphoramidase cleavage creates the monophosphate metabolite GS-331007: it may be dephosphorylated to the nucleoside metabolite, which is eliminated, or be sequentially phosphorylated into the pharmacologically active triphosphate form, which is the chain terminator of the HCV NS5B polymerase [13]. Peak plasma concentration of GS-331007 is achieved 3 h after the assumption. SOF plasma protein binding is about 61–65%. SOF absorption is not affected by food. GS-331007 is excreted in urine, with a recovery rate accounting for 66–81% of the administered dose [14].

Few data are present in the literature concerning pharmacogenetics and SOF: *ITPA* polymorphisms may still contribute to predicting anemia in patients treated with SOF plus RBV [15]. On the other hand, since the achievement of sustained virological response (SVR) with new drugs is almost 100%, the involvement of *IL28B* single nucleotide polymorphisms (SNPs) seems to be limited [16]. Our previous work suggests that stiffness, insulin resistance, baseline hemoglobin and hematocrit, and *ABCB1* gene SNPs (*3435 CT/TT* and *1236 TT* genotypes) are predictors of GS-331007 plasma concentrations at 30 days of therapy [17]. In addition, we found associations among vitamin-D-pathway-related gene polymorphisms and SOF plasma exposure and the associated hepatocarcinoma [18,19]. Concerning previous anti-HCV therapies, we suggest that different SNPs play a role in influencing telaprevir and boceprevir intracellular (IC) exposures [20,21]. Bilal et al. evaluated GS-331007 IC concentrations at day 10, highlighting higher concentrations in patients achieving SVR as compared to relapsers [22]. De Nicolo et al. developed and validated a method to quantify boceprevir and telaprevir in peripheral blood mononuclear cells (PBMCs): both drugs accumulated in the cells, and the median PBMC/plasma ratios were 28 and 5, respectively, for boceprevir and telaprevir [23].

Thus, few data are present in the literature concerning anti-HCV drug IC concentrations and pharmacogenetics, particularly for SOF, which is still used in combination with RBV in some countries, and for some critically ill patients who cannot use DAAs.

For these motives, the main objective of this study was to investigate whether variants in genes encoding transporters, and in nuclear factors involved in transporters expression regulation, could have an influence on SOF and GS-331007 IC levels at 30 days of therapy prediction, evaluating possible correlations with other drug exposures at different days.

## 2. Results

In total, 245 individuals were analyzed. The characteristics of the subjects are summarized in Table 1, including the differences between ultrasound-diagnosed cirrhotic and non-cirrhotic patients.

Considering all patients, a CHILD score was available only for 49 patients with an ecographic cirrhosis diagnosis: 37 (75.5%) were CHILD A, whereas 12 (24.5%) were CHILD B; no CHILD C patients were present. Alternatively, focusing on genotype 1 and 3 patients, a CHILD score was available only for 44 patients with an ecographic cirrhosis diagnosis: 34 (77.2%) were CHILD A, whereas 10 (22.8%) were CHILD B; no CHILD C patients were present.

**Figure 1:**
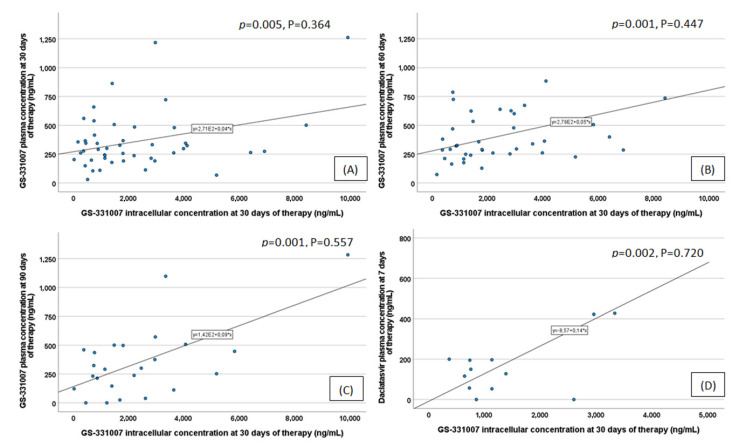
GS-331007 intracellular levels at 30 days of therapy correlated with sofosbuvir plasma concentrations at 30 days (**A**), at 60 days (**B**), at 90 days (**C**) and with daclatasvir (DAC) plasma concentrations at 7 days (**D**). *p* = *p*-value, P = Pearson coefficient.

SOF IC level evaluations at 30 days of therapy were available for only 50 patients, whereas pharmacogenetic evaluations were available for all 245. Since SOF concentrations were undetectable, only GS-331007 plasma concentrations were reported. Concerning drug exposures, several correlations were observed. Using the Pearson correlation, GS-331007 metabolite IC concentrations at 30 days significantly correlated with (*p*-value; Pearson coefficient): GS-331007 plasma concentrations at 30 days (0.005 and 0.364; Figure 1A), at 60 days (0.001 and 0.447; Figure 1B) and at 90 days (0.001 and 0.557; Figure 1C), and DAC plasma concentrations at one week (0.002 and 0.720; Figure 1D) of treatment. No correlations are suggested for the genotypes 1 and 3 group, or for RBV and LDV.

On the other hand, considering demographic and hematochemical parameters, associations with GS-331007 IC and plasma levels are summarized in Table 2 for all patients, in Table 3 for ecographic cirrhotic patients and finally in Table 4 for patients with no ecographic cirrhosis.

**Figure 2:**
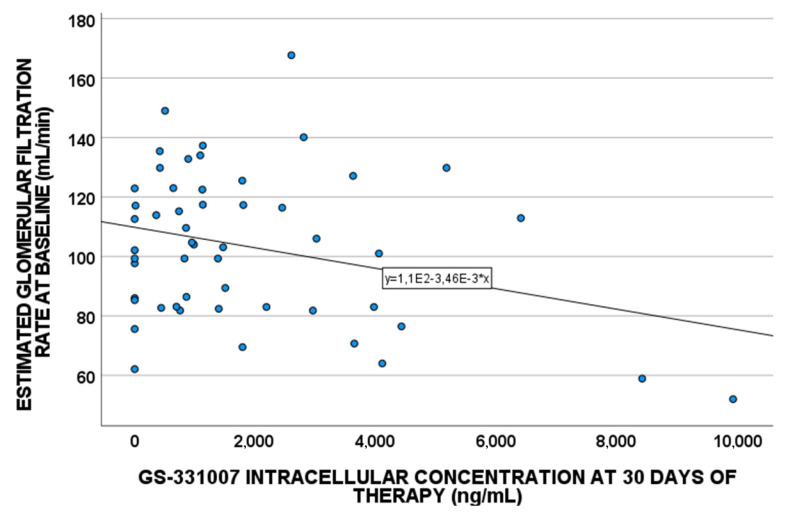
GS-331007 IC levels at 30 days of therapy correlated with baseline estimated glomerular filtration rate in all patients (*p*-value = 0.036 and Pearson coefficient = −0.291).

In particular, the estimated glomerular filtration rate (eGFR) correlated with plasma exposure in both cirrhotic and non-cirrhotic individuals, and with both plasma and IC concentration for all patients (Figure 2). No correlations are suggested for the genotypes 1 and 3 group.

**Figure 3:**
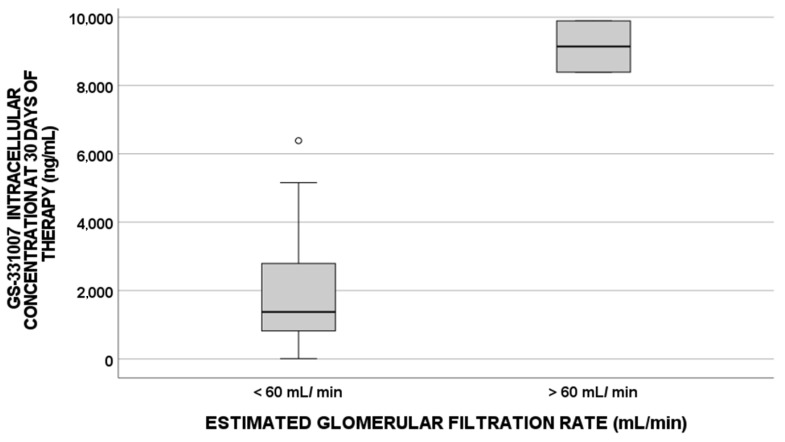
GS-331007 IC levels at 30 days of therapy correlated with the baseline estimated glomerular filtration rate cutoff value of 60 mL/min (*p*-value = 0.018, N = 48 for >60 mL/min; N = 2 for <60 mL/min).

**Figure 4:**
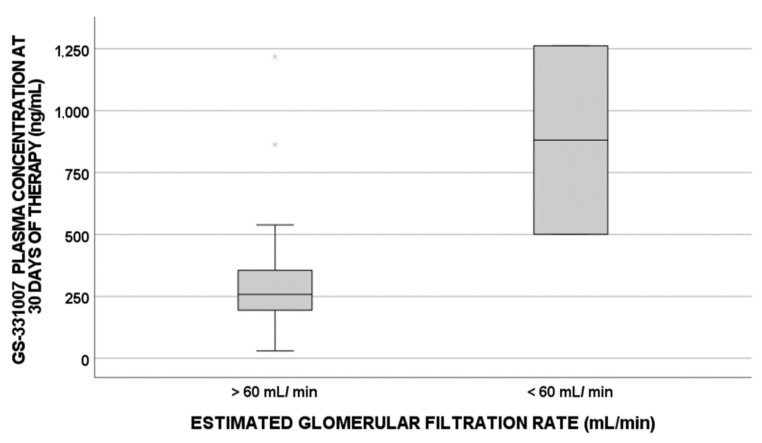
GS-331007 plasma levels at 30 days of therapy correlated with the baseline estimated glomerular filtration rate cutoff value of 60 mL/min (*p*-value = 0.032; N = 48 for >60 mL/min; N = 2 for < 60 mL/min).

For these reasons, when focusing on the eGFR cutoff value of 60 mL/min [24], which is associated with kidney impairment, a statistically significant influence was highlighted for GS-331007 IC concentrations (*p*-value = 0.018, Figure 3) and plasma concentrations (*p*-value = 0.032, Figure 4).

No associations were observed between genetic polymorphisms and GS-331007 IC exposure, when considering both all patients, and the genotypes 1 and 3 group.

In the linear multivariate regression analysis (Table 5), demographic, clinical, viral, pharmacogenetic and pharmacokinetic factors influencing metabolite IC concentrations at 30 days of therapy were evaluated: LDV treatment [*p*-value = 0.026, odd ratio (OR) 1412.863 (confidence interval (CI) 95%, 184.004; 2641.721)], baseline albumin (*p*-value = 0.010, OR, −70.093 (CI95%, −121.996; −18.00)) and baseline eGFR (*p*-value = 0.029, OR −22.677 (CI95%, −42.890; −2.464)) were significant predictors. When considering genotypes 1 and 3, LDV treatment (*p*-value = 0.048, OR 1415 (CI95%, 11.7; 2818.5)) and baseline albumin (*p*-value = 0.018, OR −71.3 (CI95%, −129.3; 13.4)) were retained in the regression model. No SNPs remained in the multivariate analyses.

## 3. Discussion

Since the viral life cycle takes place in cells, treatment effectiveness should be related to drug IC levels and their ability to pass through phospholipid membranes, penetrating different tissues. For example, anti-HIV therapy showed some limitations despite its potency, likely due to virus resistance in some compartments where antiretrovirals do not penetrate [25]. From the TDM perspective, IC drug quantification should be more representative of SOF concentrations at the active site [26]. The IC quantification of SOF and its metabolite in PBMCs represents a significant marker of drug penetration into cells, constituting a better surrogate than plasma for assessment in the hepatocellular compartment, since hepatocytes are difficult to isolate, except with a biopsy.

It is important to emphasize that IC evaluation needs to be standardized, and every association should be interpreted with caution, since IC levels do not necessarily reflect the effective unbound fraction [27]. For the first time, we analyzed SOF and GS-331007 (GS-606965 was not analyzed, since it is an intermediate analyte) metabolite IC exposure, considering the role of SNPs in affecting concentrations. Drug exposure results showed that SOF levels were undetectable in all patients, and only GS-331007 exposures were reported: this was due to extensive interconversion of SOF into its metabolite [6].

GS-331007 IC concentrations at 30 days correlated with those of plasma at 30, 60 and 90 days of therapy, despite the fact that no prediction has been observed in the final regression model. It may seem intuitive that GS-331007 IC exposure was correlated with plasma exposure, due to their direct relationship (IC penetration depends on plasma concentration), but 50 analyzed samples are likely too few to obtain a robust statistical evaluation.

Since SOF is converted into its metabolites, which are mainly eliminated by the kidneys, the role of eGFR was considered. In fact, eGFR correlated with plasma exposure in both cirrhotic and non-cirrhotic individuals but not with IC exposure (except when considering all patients): specifically, when eGFR decreased, higher plasma concentrations were detected. This was likely due to the reduced filtration rate leading to lower drug clearance and, consequently, increasing drug plasma persistence.

On the other hand, when evaluating the eGFR cutoff value of 60 mL/min, as suggested in clinical practice as a predictor of kidney function impairment, two patients showed higher IC and plasma exposures, confirming these data.

Finally, in linear multivariate regression analysis, LDV treatment, baseline albumin and eGFR were significant predictors of IC GS-331007 concentrations: albumin and eGFR were negative predictive factors, whereas LDV therapy was positive. Albumin binds to drugs; thus, it could be possible that higher albumin levels could keep GS-331007 in plasma, without allowing it to penetrate into cells [28]. Furthermore, the highlighted inverse correlation between albumin and GS-331007 IC exposure could be explained by increased hepatic activity, which could be associated with an increased drug metabolism.

Higher eGFRs were associated with lower IC GS-331007 exposure: this could be due to increased drug elimination by the kidneys, as suggested by Gil et al. [29]. On the other hand, LDV treatment may affect GS-331007 levels, since LDV and SOF share some transporters, such as ABCB1 and ABCG2 [30].

Furthermore, no SNPs remained in the final regression model: most likely because GS-331007 is not a P-gp and is perhaps a BCRP substrate instead [10,11,12,31]. However, further studies could focus on other genes encoding for metabolizing enzymes, such as carboxylesterase, which is the main protein responsible for SOF metabolism, or human cathepsin A [32]. In addition, Esposito et al. evidenced P-gp inhibitors leading to increased SOF levels [33]. Other works could aim to evaluate transporter gene SNPs, which have not been described in vivo yet: for example, organic cationic transporters 2 or organic anion transporter 1 or 3 [32].

Most anti-infective agents bind to their specific cellular targets: xenobiotics pass from the blood to extravascular sites by passive diffusion or active transport. Membrane transporters regulate drug absorption and distribution and could explain part of the inter-individual variability in drug concentration [27]. P-gp and BCRP, together with the multidrug resistance protein, are the main efflux proteins involved in drug transport: several factors, including polymorphisms in genes encoding these transporters, could affect their activity, resulting in variations in drug disposition and outcome [34]. In this study, we have not evidenced any association between pharmacogenetic markers and IC metabolite levels, likely due to the small number of patients.

The possible involvement of *IL28* SNPs should be discussed: they had a significant impact in predicting treatment outcomes in previous therapeutic regimens based on RBV and peg-IFN; in the early phase of DAAs, when SOF was added to RBV and peg-IFN, *IL28* genetic variants seemed to have a role. Yet, in recent years, their contribution has not been significant, since new DAAs are very efficient in terms of HCV eradication, without considering genetics. *IL28* gene SNPs’ impact was evaluated in this study but without yielding any significant evidence, as previously stated.

The combination of DAC/SOF/RBV treatment is known for its effectiveness, particularly when considering difficult-to-treat patients with chronic HCV infections (e.g., cirrhotics, transplant recipients and HCV-positive patients) [35].

## 4. Materials and Methods

### 4.1. Characteristics of Enrolled Patients

A total of 245 CHC patients treated with SOF (in combination with RBV and/or LDV, DAC and SIM) at the “Amedeo di Savoia” hospital (Turin, Italy) from 2014 to 2017, were analyzed in a retrospective study. The inclusion criteria were the absence of viral co-infections (hepatitis B or human immunodeficiency virus) and of major contraindications to drugs. All patients orally received 400 mg SOF once daily in combination with RBV (from 600 to 1200 mg according to body weight) as well as DAC (60 mg) or LDV (90 mg) for 12 or 24 weeks according to the concomitant drugs and HCV genotypes, as shown in the information leaflets accompanying pharmaceutical products [36,37,38]. Cirrhosis was defined as ecographic using radiology testing such as computed tomography (CT), ultrasound or magnetic resonance imaging (MRI).

The study was performed in compliance with the Declaration of Helsinki and local review board regulations; all patients gave written informed consent according to the local ethics committee’s standards (“Kinetic-C protocol,” approved by the Ethical Committee at the “A.O.U. S. Luigi Gonzaga, Orbassano, Turin,” n° 186/14 in 26/05/2015).

### 4.2. Pharmacogenetic Analyses

Genomic DNA was extracted from 191 blood samples using the MagNA Pure Compact (Roche, Monza, Italy). Alleles were assessed using a real-time polymerase chain reaction allelic discrimination system (LightCycler 96, Roche, Monza, Italy) and TaqMan allelic discrimination. Specific primers were used for the amplification of a small DNA fragment. Moreover, a polymerase with a 5′–3′ exonuclease activity was used, along with two different probes labeled with a different 5′ fluorophore (for example, FAM or VIC) and an appropriate 3′ quencher.

The gene SNPs investigated were *ABCB1* 1236 C>T (rs1128503), *ABCB1* 3435 C>T (rs1045642), *ABCB1* 2677 G>T (rs2032582), *ABCG2* 421 C>A (rs2231142), *ABCG2* 1194+928 C>A (rs13120400), *HNF4* 975 C>G (rs1884613), *IL28 860* (rs12979860) and *IL28 917* (rs8099917).

### 4.3. Pharmacokinetic Analyses

Pharmacokinetic evaluation was conducted before the new dose assumption (Ctrough). Plasma samples were isolated after whole blood centrifugation at 1400× *g* for 10 min at 4 °C. The SOF and GS-331007 concentrations in patients’ plasma were assessed using a fully validated UHPLC-MS/MS method [39]. SOF, SIM, LDV, DAC, ombitasvir, paritaprevir and ritonavir plasma exposures were evaluated at 1 and 3 days; 7 and 14 days; and 30, 60 and 90 days of therapy. IC SOF levels (N = 50) were measured at 30 days of treatment with the UHPLC-MS/MS used for plasma quantification after the application of a modified sample extraction and followed by validation.

IC quantification was performed in PBMCs, which were isolated using CPT Vacutainers (Becton, Dickinson and Co., Franklin Lakes, NJ, USA), and cell numbers and mean cell volumes were measured using an automated cell counter (Z2 Beckman Coulter, Instrumentation Laboratory, Milan, Italy), as previously described in the literature [40]. Briefly, 100 µL of patients’ plasma, spiked with internal standards, underwent a solid phase extraction (SPE) protocol with HLB 96-wells plates in order to clean up the samples.

Finally, the eluted extracts were diluted 1:1 with water and injected on a Shimadzu Nexera X2^®^ (Shimadzu, Kyoto, Japan) with an LCMS-8050 tandem mass detector: the chromatographic separation was achieved using a gradient run on a Acquity^®^ BEH C18 1.7 µm, 2.1 × 50 mm (Waters, Milan, Italy) UHPLC column, for 5 min for each sample. We evaluated SOF, SIM, LDV, DAC, ombitasvir, paritaprevir and ritonavir plasma concentrations at 1 and 3 days; 7 and 14 days; and 30, 60 and 90 days of therapy; IC SOF levels were measured at 30 days of treatment.

### 4.4. Statistical Analyses

The Shapiro–Wilk test was used to test normality. Non-normal variables were summarized as median values and interquartile range (IQR); dichotomic variables were summarized as numbers and percentages. All genetic variants were evaluated for Hardy–Weinberg equilibrium using the χ^2^ test for determining the observed genotype frequencies. Kruskal–Wallis and Mann–Whitney tests were used to test differences in continuous variables between genetic groups, considering that the level of statistical significance (*p*-value) was <0.05. Correlations among drug concentrations at different days were evaluated using the Pearson test. In conclusion, the predictive capability of the investigated variables was assessed using univariate (*p*-value < 0.2) and multivariate (*p*-value < 0.05) linear regression analysis. IBM SPSS Statistics software 27.0 for Windows (Chicago, Illinois, USA) was used.

## 5. Conclusions

This is the first study reporting data on IC evaluation in a cohort of patients treated with new anti-HCV drugs, specifically SOF and its metabolite. Our work shows that GS-331007 IC exposure can be predicted not only by hematochemical parameters (eGFR and albumin) but also in the use of LDV treatment. Further studies in larger and different cohorts of patients are needed to better understand these preliminary data.

Analyses of SOF pharmacogenetic and pharmacokinetic IC profiles are lacking in the literature; therefore, future studies focusing on other gene SNPs are required to clarify these aspects.

Nevertheless, this work may represent a starting point for better management of the administration of SOF, in order to limit RBV toxicity when it is co-administered.

Since new anti-HCV drugs have an efficiency rate of 99%, these data could be useful for regions that currently use these new drugs with RBV; in addition, these results could be the basis for the understanding of IC exposure for similar drugs in the future.

## Figures and Tables

**Table 1:** Patients’ characteristics at baseline. Differences between cirrhotic and non-cirrhotic patients are reported with *p*-values.

Characteristics	All Cases		Genotypes 1 and 3		No Ecographic Cirrhosis Diagnosis		Ecographic Cirrhosis Diagnosis		Statistical Significance (*p*-Value)
*n*	245		201		183		62		/
Age (year), median (IQR)	51 (48–60)		53 (48–62)		54 (48–63)		51.5 (41.9–60.2)		0.125
Male sex, n (%)	188 (76.7)		156 (77.6)		135 (73.8)		53 (85.5)		0.081
BMI (Kg/m^2^), median (IQR)	26 (24–30)		25.7 (23.5–28.5)		26.8 (23.8–30)		25.9 (24.4–32.8)		0.297
HCV-RNA (Log IU/mL), median (IQR)	6.5 (5.7–6.9)		6.3 (5.8–6.7)		3.38 (3.07–4.07)		3.64 (3.3–4.5)		0.033
Cryoglobulinemia, n (%)	86 (35.1)		67 (33.3)		56 (30.6)		30 (48.4)		0.014
Insulin resistance, n (%)	28 (11.4)		21 (10.4)		15 (8.2)		13 (21)		0.010
Metavir score, n (%)	F0–F3 F4	114 (46.5) 131 (53.5)	F0–F3 F4	88 (43.8) 113 (56.2)	F0–F3 F4	108 (59) 75 (41)	F0–F3 F4	6 (9.7) 56 (90.3)	<0.001 <0.001
Alanine-aminotransferase (IU/mL), median (IQR)	78.5 (58.7–95.7)		80 (47–119)		86 (71.5–213)		91.5 (51–128.)		0.295
Albumin (g/L), median (IQR)	39.5 (31.7–43)		40 (36–43)		41 (39.5–45)		37.5 (32–43)		<0.001
Hemoglobin (g/dL), median (IQR)	14.7 (13.6–16.4)		15.1 (14.3–16.0)		15.2 (14.2–16.1)		14.6 (13.2–15.7)		<0.004
Hematocrit (%), median (IQR)	43.5 (40–46.8)		44 (41.3–46.1)		44.3 (41.7–46.6)		41.9 (38.8–45.2)		<0.004
Mean corpuscular volume (fL), median (IQR)	88.7 (84.3–93.5)		89 (86.4–92.6)		89.6 (86.3–92)		88.2 (87–93)		0.038
Estimated glomerular filtration rate (mL/min), median (IQR)	104.8 (82.5–121.2)		101 (84.4–116.1)		99.3 (88.1–108)		100.2 (66.9–124)		0.638
Hepatocarcinoma n (%)	4 (1.6)		4 (2)		0		4 (6.5)		NC
Patients treated with ribavirin, n (%)	85 (34.7)		61 (30.3)		67 (36.6)		18 (29)		0.281
Patients treated with daclatasvir, n (%)	62 (25.3)		55 (24.7)		39 (21.3)		23 (37.1)		0.018
Patients treated with ledipasvir, n (%)	66 (26.9)		63 (31.3)		42 (23)		24 (38.7)		0.020
Median GS-331007 plasma concentrations at 1 month of therapy (ng/mL), median (IQR)	274 (198–367)		335 (239.3–490.8)		298.5 (198.7–459.5)		243 (184–356)		0.273
Median GS-331007 intracellular concentrations at 1 month of therapy (ng/mL), median (IQR)	1118.8 (355.9–2794.5)		1126.1 (416.2–2937.9)		799.5 (10.5–2293.7)		1126.5 (690.3–5155.8)		0.086
Median GS-331007 plasma/intracellular concentrations at 1 month of therapy, median (IQR)	0.1 (0.07–0.2)		0.21 (0.08–0.57)		0.22 (0.1–0.6)		0.14 (0.05–0.2)		0.048
Median daclatasvir concentrations at 1 month of therapy (ng/mL), median (IQR)	182 (104–294)		176.5 (93.8–277.0)		177 (88.2v314.7)		203 (149.5v294)		0.475
Median ledipasvir concentrations at 1 month of therapy (ng/mL), median (IQR)	232 (172–329)		235.5 (158.5–332.3)		227.5 (157.5–328.2)		232 (187.5–412)		0.560
Genotype, n (%)	1	158 (64.5)	1	158 (78.6)	1	119 (65)	1	39 (62.9)	0.048
	2	12 (4.9)			2	10 (5.5)	2	2 (3.2)	
	3	43 (17.6)	3	43 (21.4)	3	26 (14.2)	3	17 (27.4)	
	4	32 (13.1)			4	28 (15.3)	4	4 (6.5)	

**Table 2:** Associations of GS-331007 intracellular (IC) and plasma levels with demographic and hematochemical parameters in all patients.

Variables in All Patients	GS-331007 Intracellular Concentration at 30 Days (ng/mL) [*p*-Value; Pearson Coefficient]	GS-331007 Plasma Concentration at 30 Days (ng/mL) [*p*-Value; Pearson Coefficient]
Age (year)	<0.001; 0.412	0.046; 0.187
Estimated glomerular filtration rate (mL/min)	0.036; −0.291	<0.001; −0.447
Baseline hemoglobin (g/dL)	<0.001; −0.531	0.035; −0.200
Baseline hematocrit (%)	0.001; −0.465	/
Mean Corpuscular Volume (fL)	/	/
Albumin (g/L)	0.020; −0.313	/
BMI (Kg/m^2^)	/	/
Alanine-aminotransferase (IU/L)	/	/
HCV-RNA (Log IU/mL)	/	/

**Table 3:** Associations of GS-331007 IC and plasma levels with demographic and hematochemical parameters in ecographic cirrhotic patients.

Variables in Ecographic Cirrhotic Patients	GS-331007 Intracellular Concentration at 30 Days (ng/mL) [*p*-Value; Pearson Coefficient]	GS-331007 Plasma Concentration at 30 Days (ng/mL) [*p*-Value; Pearson Coefficient]
Age (year)	/	/
Estimated glomerular filtration rate (mL/min)	/	0.001; −0.572
Baseline hemoglobin (g/dL)	0.001; −0.726	/
Baseline hematocrit (%)	0.007; −0.667	/
Mean Corpuscular Volume (fL)	/	/
Albumin (g/L)	/	/
BMI (Kg/m^2^)	/	/
Alanine-aminotransferase (IU/L)	/	/
HCV-RNA (Log IU/mL)	/	/

**Table 4:** Associations of GS-331007 IC and plasma levels with demographic and hematochemical parameters in patients with no ecographic cirrhosis.

Variables in Patients with No Ecographic Cirrhosis	GS-331007 Intracellular Concentration at 30 Days (ng/mL) [*p*-Value; Pearson Coefficient]	GS-331007 Plasma Concentration at 30 Days (ng/mL) [*p*-Value; Pearson Coefficient]
Age (year)	0.002; 0.426	0.006; 0.308
Estimated glomerular filtration rate (mL/min)	/	0.001; −0.403
Baseline hemoglobin (g/dL)	0.015; −0.338	0.039; −0.238
Baseline hematocrit (%)	/	/
Mean Corpuscular Volume (fL)	/	/
Albumin (g/L)	/	/
BMI (Kg/m^2^)	/	/
Alanine-aminotransferase (IU/L)	/	/
HCV-RNA (Log IU/mL)	/	/

**Table 5:** Linear regression analyses: parameters able to predict GS-331007 metabolite ICs at 1 month of therapy. BMI: body mass index; ALT: alanine aminotransferase; eGFR: estimated glomerular filtration rate.

	GS-331007 Metabolite Intracellular Concentrations at 30 Days of Therapy			
	UNIVARIATE		MULTIVARIATE	
	*p*-Value	OR (95% IC)	*p*-Value	OR (95% IC)
BMI at baseline	0.320	52.476 (−52.346; 157.298)		
Age	<0.001	96.995 (52.222; 141.769)	0.595	−27.978 (−135.863; 79.906)
Gender	0.010	1778.108 (445.416; 3110.801)	0.248	947.016 (−697.439; 2591.471)
Cirrhosis	0.147	895.063 (−322.849; 2112.974)	0.539	372.594 (−864.776; 1609.965)
Metavir score	0.482	288.911 (−528.453; 1106.274)		
Ribavirin treatment	0.081	−977.127 (−2077.495; 123.240)	0.396	552.134 (−769.093; 1873.361)
Daclatasvir treatment	0.165	−856.788 (−2076.670; 363.095)	0.293	623.597 (−570.200; 1817.394)
Ledipasvir treatment	0.002	1685.431 (660.043; 2710.820)	0.026	1412.860 (184.004; 2641.721)
Baseline ALT	0.387	−2,445 (−8.062; 3.171)		
Baseline eGFR	0.005	−34.696 (−58.224; −11.169)	0.029	−22.677 (−42.890; −2.464)
Baseline albumin	0.046	−60.238 (−119.492; −0.985)	0.010	−70.093 (−121.996; −18.190)
GS-331007 plasma concentrations at 30 days of therapy	0.022	2.936 (0.452; 5.421)	0.347	−1.774 (−5.583; 2.035)
*ABCB1 3435 TT*	0.144	−901.457 (−2119.030; 316.116)	0.738	289.774 (−1495.686; 2075.233)
*ABCB1 1236 TT*	0.017	−1554.498 (−2818.099; −290.898)	0.465	−548.368 (−2074.326; 977.591)
*ABCB1 2677 TT*	0.076	1053.002 (−112.565; 2218.569)	0.139	942.688 (−324.899; 2210.275)
*ABCG2 421 CA*	0.606	514.735 (−1472.017; 2501.487)		
*ABCG2 1194 + 928 TC/CC*	0.353	522,128 (−595,589; 1639,845)		
*HNF4α 975 CG/GG*	0.169	−798.028(−1945.495; 349.440)	0.552	−366.867 (−1627.415; 893.680)
*IL 860 TT*	0.329	586.04 (−505;1477)		
*IL 917 CC*	0.484	−345.9 (−1330.9;639.9)		

## Data Availability

Data was contained in the manuscript.
